# Prebiotic Interconversion of Pyruvate and Lactate over Zeolite‐Supported Ni Catalyst

**DOI:** 10.1002/anie.202503747

**Published:** 2025-04-21

**Authors:** Youngdong Song, Eko Budiyanto, Ashwani Kumar, Gautier Landrot, Harun Tüysüz

**Affiliations:** ^1^ Department of Heterogeneous Catalysis Max‐Planck‐Institut für Kohlenforschung Kaiser‐Wilhelm‐Platz 1 45470 Mülheim an der Ruhr Germany; ^2^ Synchrotron SOLEIL L'Orme des Merisiers Départementale 128 Saint‐Aubin 91190 France; ^3^ Catalysis and Energy Materials IMDEA Materials Institute Calle Eric Kandel 2 Getafe Madrid 28906 Spain

**Keywords:** Heterogeneous catalysis, Hydrothermal vents, Nickel, Origin of life, Zeolites

## Abstract

Submarine hydrothermal vents harbor diverse microbial communities and have long intrigued researchers studying the origin of life. Transition metals in these environments can be reduced by serpentinization, potentially forming zeolite‐supported transition metal nanoparticles capable of driving prebiotic chemistry. This inorganic structure could catalyze biochemical reactions, including converting metabolically crucial pyruvate before the emergence of biological processes. This study explores the catalytic interconversion of pyruvate and lactate, mediated by lactate dehydrogenase in biochemical systems, using inorganic zeolite Y‐supported Ni nanoparticles (Ni/Y) under mild hydrothermal vent conditions. Our results demonstrate that Ni/Y effectively catalyzes the hydrogenation of pyruvate in an inert environment, facilitated by the in situ generation of H₂ through an autocatalytic reaction between Ni/Y and H₂O. Post‐reaction analysis by X‐ray absorption spectroscopy (XAS) revealed structural transformations in the catalyst, including the formation of unique nickel oxide and hydroxide species, along with extra‐framework aluminum from zeolite dealumination, resulting in a thin amorphous nickel oxide/hydroxide layer. Notably, Ni/Y also enables the oxidative reconversion of lactate to pyruvate under atmospheric conditions—an essential reaction catalyzed by lactate dehydrogenase in biological systems. These findings underscore the potential prebiotic role of Ni/Y, suggesting they may have catalyzed the synthesis of key metabolic intermediates.

## Graphical abstract

Synthetic zeolite Y‐supported Ni mineral can catalyze the interconversion of metabolically crucial pyruvate and lactate under hydrothermal vent conditions, which could pave the way for the modern biological process by lactate dehydrogenase and nicotinamide adenine dinucleotide.

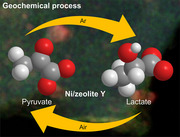

## Introduction

Deep‐sea hydrothermal vents have garnered significant interest from the origin of life community due to their favorable conditions for chemical evolution.^[^ ^1^, ^2^, ^3^, ^4^, ^5^ ^]^ Hydrothermal vents occur when seawater seeps through fractures in the ocean floor. The heated seawater reacts with underlying rocks, producing an outflow rich in reduced chemical species such as H_2_S, Fe^2+^, and CH_4_.^[^ ^6^ ^]^ When this hot and chemically rich fluid is mixed with cold (2 °C) and oxidizing seawater, it forms hydrothermal vent chimneys that possess a redox potential.^[^ ^7^ ^]^ This chemical disequilibrium of hydrothermal vents creates a unique ecosystem that harnesses chemical energy through a process called chemosynthesis.^[^ ^8^ ^]^ Chemosynthetic microbes, such as methanogens and acetogens, thrive in these environments, utilizing H_2_ and CO_2_ for their metabolism.^[^ ^9^, ^10^, ^11^ ^]^ This has inspired the hypothesis that life's origin may have originated at hydrothermal vents since their discovery in 1977.^[^ ^12^ ^]^

Hydrothermal vents are broadly categorized into two types based on their characteristics. Black smokers are located directly above magma chambers and emit fluids at temperatures up to 400 °C with acidic pH (2–3) due to sulfide‐rich minerals.^[^ ^1^, ^2^, ^3^, ^4^ ^]^ In contrast, white smokers located off ridges release fluids at lower temperatures ranging from 40° to 116 °C with basic pH (9–11) attributed to carbonate minerals.^[^ ^1^, ^2^, ^3^, ^4^, ^13^ ^]^ The chemistry at hydrothermal vents varies depending on the geological setting. In particular, serpentinization, a process in which ultramafic minerals react with water, plays a key role in the formation of alkaline hydrothermal systems such as white smokers. Typically, the reaction of iron(II) containing olivine mineral with seawater produces serpentine and H_2_.^[^ ^14^ ^]^ The continuous supply of H_2_ (10–16 mmol kg^−1^), combined with the exothermic nature of serpentinization, creates reductive conditions favorable for the emergence of life at hydrothermal vents.^[^ ^15^ ^]^

Zeolite‐supported Ni has been extensively investigated as a catalyst for hydrogenation due to its ability to split H_2_ molecules.^[^ ^16^, ^17^, ^18^, ^19^, ^20^ ^]^ In hydrothermal vents, Ni exists as native metals (Ni°) or alloys like NiFe and NiFeS, indicating that serpentinization‐induced reduction of Ni can occur under hydrothermal vent conditions.^[^ ^21^ ^]^ Ni is also present in methanogenic archaea inhabiting hydrothermal vents, which utilize Ni‐containing enzymes such as [NiFe]‐hydrogenase, carbon monoxide dehydrogenase, acetyl‐coenzyme A (acetyl‐CoA) synthase/decarbonylase, and methyl‐coenzyme M reductase to thrive in the conversion of H_2_ and CO_2_ into CH_4_.^[^ ^22^, ^23^ ^]^ These microorganisms date back to 3.42 billion years ago and may have utilized Ni in their metabolic pathways, suggesting that geochemical reactions involving Ni could have played a significant role in early biochemical processes.^[^ ^22^, ^24^ ^]^ In addition, zeolites, which are synthesized under hydrothermal conditions, are crystalline microporous aluminosilicates that occur naturally in hydrothermal vents through the reaction of volcanic rocks under high pressure and temperature.^[^ ^25^, ^26^, ^27^ ^]^ While zeolites have been identified in certain hydrothermal vent systems, there are currently no direct reports of their occurrence in serpentinizing hydrothermal environments. However, their presence in hydrothermal settings still supports their potential relevance in prebiotic chemistry. Their high porosity allows molecules to accumulate within their structure, potentially facilitating the abiogenic synthesis of organic molecules that are crucial for the emergence of life.^[^ ^28^ ^]^ This synergy between Ni and zeolite under hydrothermal vent conditions implies their potential role in early geochemical reactions, leading to the conversion of metabolic intermediates such as pyruvate.

Pyruvate (CH_3_COCOO^−^) is the conjugate base of the simplest α‐keto acid, playing a central role in biochemistry.^[^ ^29^ ^]^ Under anaerobic conditions, such as those found in hydrothermal vents, pyruvate can be converted to acetyl‐CoA by either pyruvate‐formate lyase or pyruvate‐ferredoxin oxidoreductase.^[^ ^30^, ^31^ ^]^ Another key reaction involving pyruvate is its interconversion with lactate, catalyzed by lactate dehydrogenase (LDH) (Equation 1). In this process, pyruvate and lactate are interconverted by the ubiquitous LDH, either with or without the involvement of nicotinamide adenine dinucleotide (NAD).^[^ ^32^ ^]^ While this reaction is typically associated with heterotrophic metabolism, its fundamental chemical transformation suggests that it could have prebiotic relevance. The widespread presence of LDH does not necessarily imply an ancient origin, but the abiotic interconversion of pyruvate and lactate under hydrothermal conditions remains an important consideration. Given that the synthesis of pyruvate from CO₂ has been reported under simulated hydrothermal conditions, further investigation of its conversion by transition metals is essential to bridge the gap between prebiotic chemistry and biology.^[^ ^33^, ^34^, ^35^ ^]^

(1) $$\begin{array}{cc} & CH_{3}\mathrm{COCO}O^{-}\left(\mathrm{pyruvate}\right)+H_{2}\\ & ⇌CH_{3}\mathrm{CHOHCO}O^{-}\left(\mathrm{lactate}\right) \end{array}$$

Novikov and Copley studied the conversion of pyruvate into various products such as lactate, thiolacetate, thiolacetate disulfide, and propionate under mild hydrothermal vent conditions of 25° and 110 °C.^[^ ^36^ ^]^ Under different gas environments containing H_2_, H_2_S, CO_2_, and NH_4_Cl, they found that pyruvate can be converted into a mixture of products using naturally occurring transition metal sulfide minerals as catalysts, including pyrrhotite (Fe_1−x_S) and troilite (FeS). Together with our collaborator, we reported the conversion of pyruvate to citramalate at room temperature under atmospheric conditions using Ni_3_Fe nanoparticles.^[^ ^37^ ^]^ The catalyst was able to convert pyruvate to parapyruvate as an intermediate via homo‐aldol condensation, which was further converted into citramalate by decarboxylation. De Aldecoa et al. studied the interconversion of pyruvate and lactate using natural pyrrhotite mineral as a catalyst.^[^ ^38^ ^]^ This natural mineral did not convert pyruvate under inert conditions, while pyruvate was converted to lactate in the presence of S° and/or H_2_S. The conversion of lactate to pyruvate was also investigated using the same mineral as a catalyst, showing that pyrrhotite and S° are necessary for lactate conversion. However, the natural pyrrhotite contains other minerals, including chalcopyrite, pyrite, and chlorite, making it difficult to identify the active surfaces. Schlikker et al. reported the conversion of pyruvate to lactate using nickel powder as a catalyst under mild hydrothermal vent conditions.^[^ ^39^ ^]^ Their study demonstrated that 20 mM of pyruvate could be converted to 18.5 mM of lactate at 60 °C over 72 h under a 5 bar H_2_ environment without pyridoxal. The addition of pyridoxal reduced the lactate concentration to 13.6 mM under this condition, as it favors reductive amination. Sugiyama et al. demonstrated the oxidative dehydrogenation of lactate to pyruvate using Pd/C catalysts in an aqueous phase.^[^ ^40^ ^]^ They showed that the pyruvate yield reached 63.9% at 85 °C under 7.5 bar of O_2_. The addition of 0.19 wt % Te promoted the yield of pyruvate up to 68.2%. Yin et al. reported the conversion of lactate to pyruvate in a gas phase with P‐doped Fe─Mo bimetallic oxide catalysts.^[^ ^41^ ^]^ Compared to monometallic oxides, enhanced activity was observed in bimetallic Fe─Mo oxides due to the formation of Fe─O_x_─Mo solid solution. This resulted in novel redox and acid–base properties, which are favorable for the oxidative dehydrogenation of lactate. However, the interconversion of pyruvate and lactate using zeolite‐supported Ni under mild hydrothermal vent conditions has not been systematically investigated in the context of the origin of life.

Herein, we investigate the catalytic interconversion of pyruvate and lactate using a zeolite Y‐supported Ni (Ni/Y) catalyst under simulated mild hydrothermal vent conditions, aiming to bridge the gap between geochemical and biological processes. The catalytic activity for pyruvate conversion was systematically evaluated by varying reaction temperature and time. Control experiments and post‐reaction analyses provide key insights into the plausible reaction mechanism. Additionally, the synthetic mineral's ability to facilitate the oxidation of lactate to pyruvate under atmospheric conditions was examined, highlighting its parallel to the modern biological process catalyzed by LDH and NAD⁺.

## Results and Discussion

To explore the interconversion of pyruvate and lactate under mild hydrothermal vent conditions, a Ni/Y catalyst was prepared by the wet impregnation method using commercial zeolite Y─H (Si/Al = 2:55) as the support. The Ni loading was maintained at 10 wt % throughout the study to prevent the agglomeration of Ni nanoparticles and to ensure sufficient liquid products for analysis. As shown in Figure S1, the X‐ray diffraction (XRD) pattern of the calcined powder exhibited the reflections of zeolite Y (JCPDS No. 01–077–1551) and NiO (JCPDS No. 00–047–1049). The calcined catalyst was further reduced under the flow of H_2_ at 400 °C, which is the maximum temperature of hydrothermal vent fluid. The XRD pattern of the reduced catalyst showed characteristic reflections from zeolite Y and metallic Ni (JCPDS No. 00–004–0850) without the presence of any NiO reflections. It was noted that the diffraction patterns of zeolite Y remained unchanged, confirming that its crystal structure was retained during catalyst preparation.

**Figure 1:**
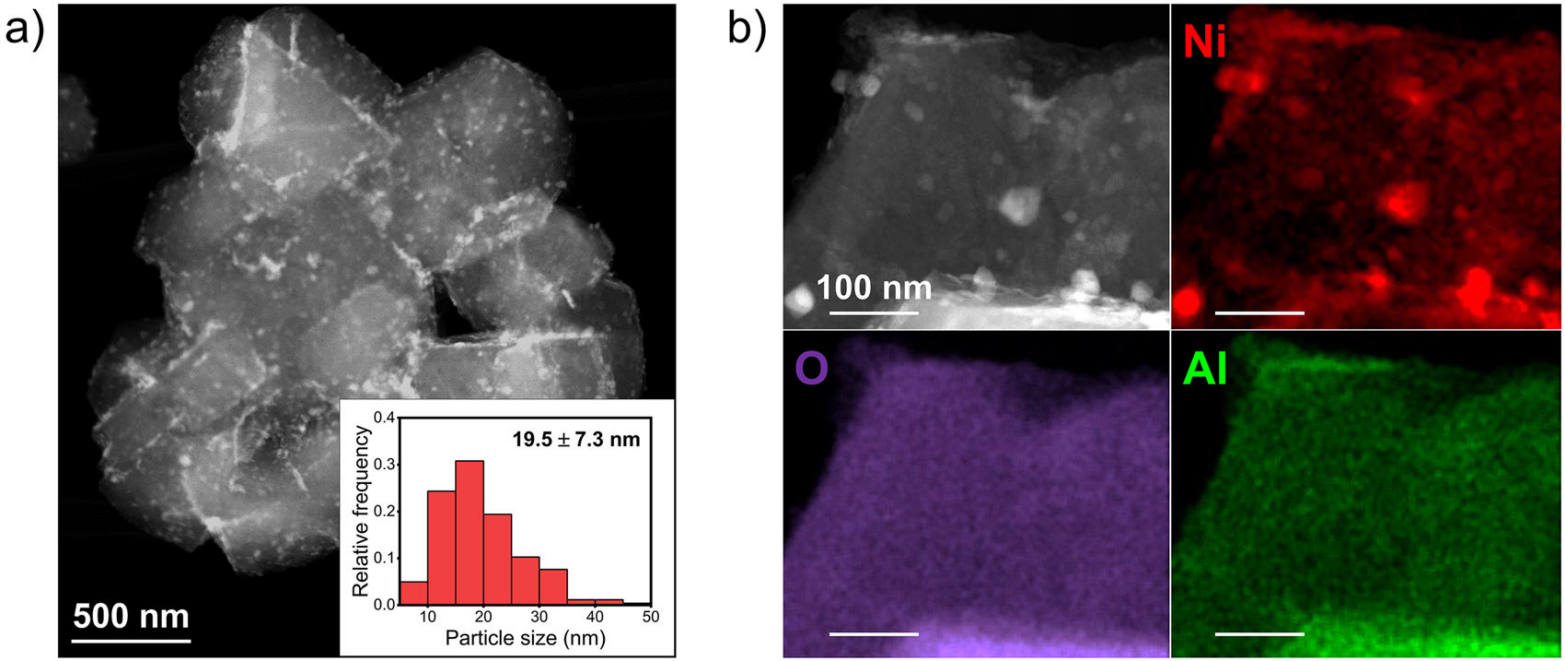
a) HAADF‐STEM image and b) EDX elemental mapping of fresh Ni/Y catalyst. The inset of a) shows the particle size distribution.

High‐angle annular dark‐field scanning transmission electron microscopy (HAADF‐STEM) was used to gain deeper insight into the particle size and the dispersion of Ni nanoparticles on zeolite Y. As shown in Figure 1, it was revealed that the Ni nanoparticles were dispersed on zeolite Y support with an average particle size of 19.5 nm. In Figure 1b, energy‐dispersive X‐ray spectroscopy (EDX) elemental mapping at a higher magnification revealed that the Ni nanoparticles were homogeneously dispersed on zeolite Y. It was found that the Ni loading was measured to be 10.7 wt %, which corresponds to the targeted loading. It has also been observed that some nickel nanoparticles are too small to be accurately measured in particle size due to the ion exchange property of zeolite Y with Ni^2+^ cation. Protons in zeolite Y can be exchanged with Ni^2+^ during the impregnation step, resulting in a high dispersion of Ni nanoparticles on zeolite Y.^[^ ^42^, ^43^ ^]^

**Figure 2:**
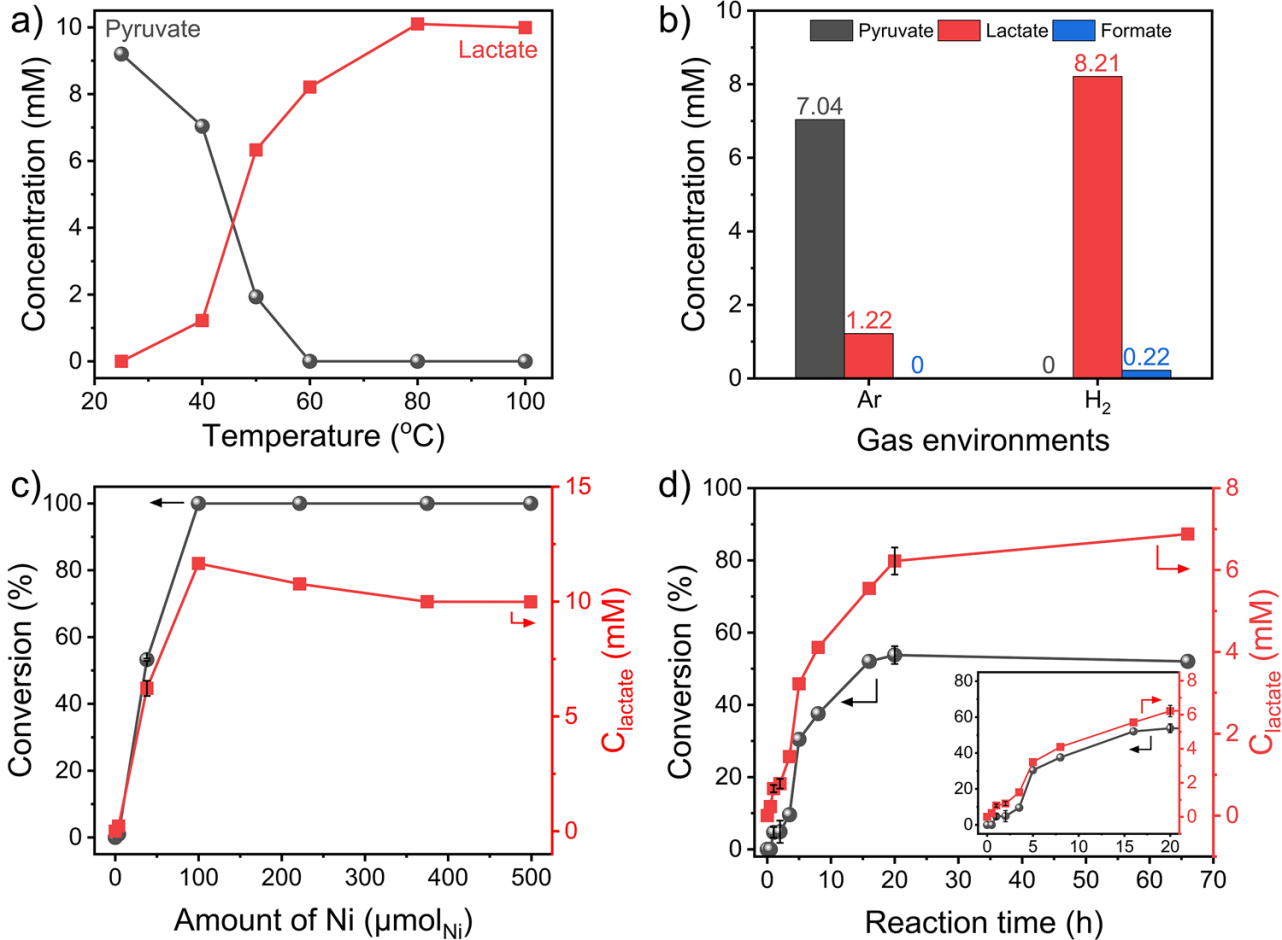
Catalytic performance of Ni/Y under mild hydrothermal vent conditions. a) The catalytic activity of Ni/Y (500 µmol_Ni_) as a function of temperature (13 mM_pyruvate_, 10 bar_Ar_, 20 h). b) Effect of gas environment on pyruvate and lactate concentrations (500 µmol_Ni_, 13 mM_pyruvate_, 40 °C, $10\;{\text{bar}}_{\text{Ar or}\;H_{2}}$, 20 h). (c) Catalytic performance as a function of Ni/Y amount (100 °C, 10 bar_Ar_, 20 h). (d) Time‐resolved conversion and lactate concentration (38 µmol_Ni_, 13 mM_pyruvate_, 10 bar_Ar_, 100 °C). The inset shows a magnified view of the first 20 h. Black arrows indicate conversion, while red arrows represent lactate concentration. Error bars denote the standard deviation from triplicate measurements.

After structural characterization, the catalyst was investigated for the conversion of pyruvate under mild hydrothermal vent conditions. Ni─Mo alloy autoclaves with Ti liners were used to simulate hydrothermal vent conditions, preventing undesired side reactions from the Ni─Mo alloys and contamination from other carbon sources, such as commonly used polytetrafluoroethylene liners. Blank tests confirmed the products could only be produced from the conversion of pyruvate in the presence of Ni/Y. Firstly, the effect of reaction temperatures on pyruvate conversion was investigated in a temperature range of the Lost City hydrothermal field (LCHF), a representative white smoker. As seen in Figure 2a, pyruvate was converted to lactate as low as 40 °C. The detection of lactate at even the low temperature of 40 °C indicated that pyruvate was hydrogenated to lactate under the simulated conditions of the LCHF in the presence of a synthetic Ni/Y catalyst. The lactate concentration increased up to 80 °C with the decrease in pyruvate concentration, implying that higher temperatures are preferred in LCHF for the catalytic hydrogenation of pyruvate to lactate. The carbon balance was calculated to be approximately 70%–80%, which is due to the zeolite's highly microporous nature and strong adsorption capacity. It was observed that lactate concentration increased without pyruvate in the temperature range of 60°–80 °C, which could reflect the desorption of lactate from highly microporous zeolite Y. Notably, the hydrogenation of pyruvate occurs under inert Ar environments, meaning that hydrogen is provided from H_2_O.

To further investigate the role of Ni/Y in the reaction, more control experiments were carried out. As shown in Figure S2, pyruvate was not converted to lactate without the Ni/Y catalyst under H_2_, indicating that the hydrogenation of pyruvate is a catalytic process and Ni/Y acts as a catalyst. When the corresponding amount of zeolite Y (264 mg) was used for the reaction at 25 °C under an Ar atmosphere, lactate was not detected with a slight decrease in pyruvate concentration to 11.13 mM. This indicates that zeolite Y might adsorb pyruvate due to its microporosity.^[^ ^44^ ^]^ Increasing the reaction temperature to 100 °C with zeolite Y without Ni showed marginal lactate formation (0.33 mM) compared to the Ni/Y catalyst that showed full conversion of pyruvate to lactate. These control experiments confirmed that Ni/Y plays a catalytic role in the hydrogenation of pyruvate to lactate. To compare the effect of the gas environment, a control experiment was performed in an H_2_ environment, which showed 100% conversion of pyruvate to lactate and formate, as shown in Figure 2b. This supports in situ H_2_ formation from H_2_O under the Ar environment and the subsequent hydrogenation of pyruvate in the presence of Ni/Y catalyst.^[^ ^45^ ^]^ This also implies that H_2_O could react with Ni/Y for the in situ H_2_ generation as low as 40 °C, since a control experiment without Ni/Y showed no lactate formation.

Next, the amount of catalyst was screened for the optimization of reaction conditions. As seen in Figure 2c, the conversion reaches 100% from 100 µmol_Ni_ and remains at 100% with higher amounts of catalyst. However, more than 100 µmol_Ni_ resulted in a decrease in lactate concentration from 11.7 to 9.9 mM, which could be attributed to the adsorption of lactate on the catalyst surface. To confirm the adsorption of lactate on the catalyst, the adsorption capacity of zeolite Y was further investigated since zeolites are mainly responsible for the adsorption of molecules due to their high degree of microporosity. As shown in Figure S3, the proportionality of lactate adsorption with the amount of zeolite Y confirmed the adsorption of lactate on zeolite Y. The corresponding amount of zeolite in the catalyst containing 500 µmol_Ni_ was 264 mg, where the lactate concentration decreased from 12.5 to 9.4 mM. This implies that 9.3 µmol of lactate was adsorbed on zeolite Y.

As a next step, the 38 µmol_Ni_ was chosen to minimize the amount of catalyst and to investigate the time‐dependent reaction profile. As shown in Figure 2d, the conversion and lactate concentration showed a gradual increase over 5 h and then reached a plateau of 54% after 20 h. Despite the extension of the reaction time, the conversion remained at 54% for 65 h, suggesting either catalyst deactivation or the depletion of in situ generated H_2_. To investigate the intermediates and byproducts, we carried out an isotope‐labeled reaction using 2‐^13^C‐sodium pyruvate. As shown in Figure S4, the ^13^C NMR spectrum revealed that lactate is the only product. This indicates that lactate selectivity is 100% and that the reaction is purely a matter of conversion. The pressure effect was also studied by increasing the reaction pressure to 50 bar under an Ar environment, but the pressure did not affect the conversion of pyruvate to lactate (Figure S5). Moreover, the observation of a sigmoidal curve in Figure 2d is a characteristic fingerprint of the autocatalytic process, which plays a crucial role in the origin of life.^[^ ^46^, ^47^ ^]^

The hydrogenation of pyruvate requires H_2_, which can be provided by the reaction of Ni and H_2_O via an autocatalytic process. The oxygen species produced during the process are bonded to the catalyst by forming metal oxides or hydroxides. The control experiment with zeolite Y without Ni nanoparticles did not show any noticeable conversion or concentration of lactate, indicating that Ni is mainly responsible for the in situ H_2_ production. According to the results, Ni reacts with H_2_O to produce in situ H_2_ and NiO (Equation 2).^[^ ^48^ ^]^

(2) $$\mathrm{Ni}+H_{2}O\to \mathrm{NiO}+H_{2}$$

**Figure 3:**
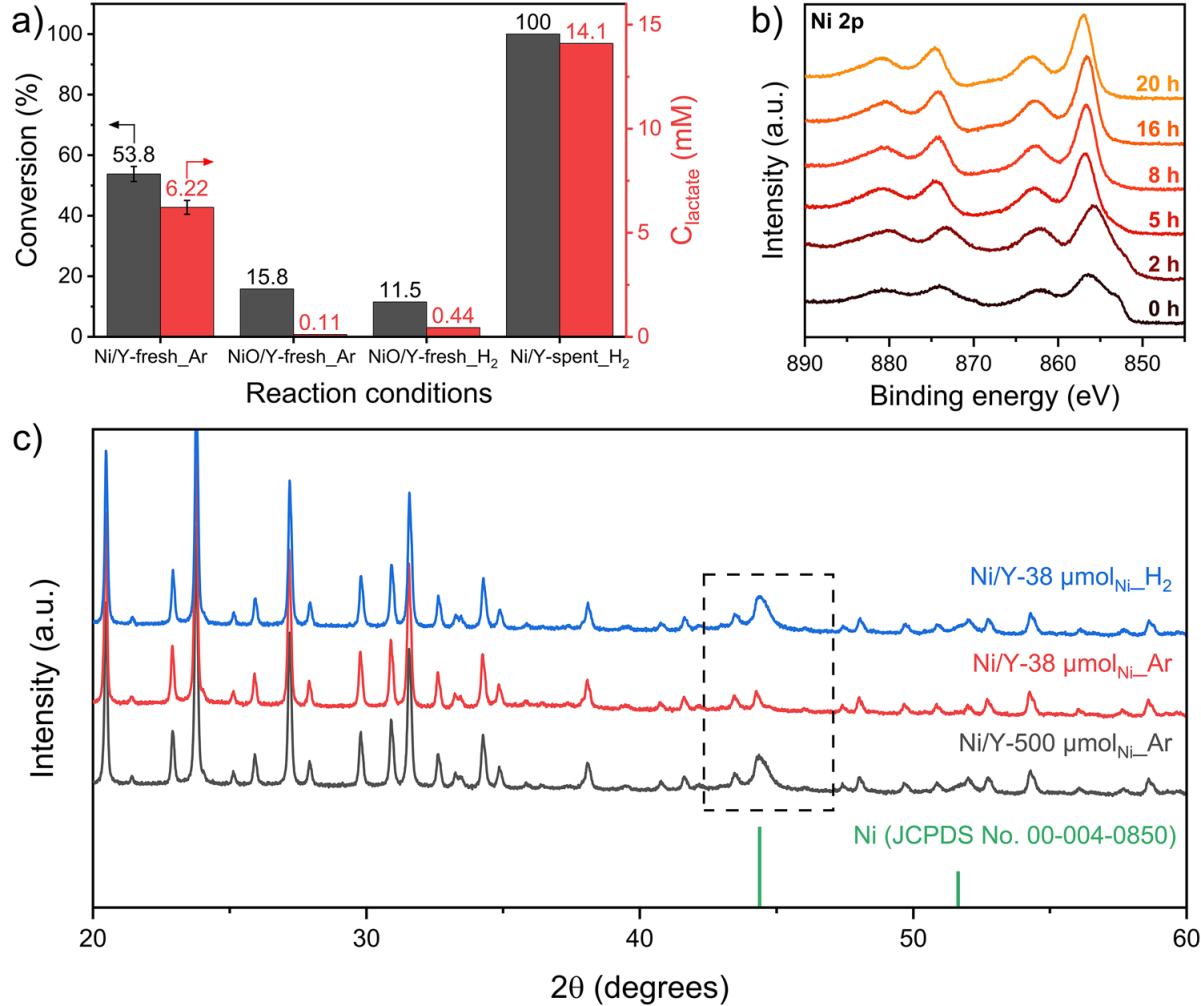
a) Control experiments for catalyst deactivation and active phase investigation (38 µmol_Ni_, 100 °C, 10 bar, 20 h). b) Time‐resolved XPS spectra of Ni 2p for 20 h under 10 bar_Ar_ using 38 µmol_Ni_. c) The XRD patterns of spent catalysts under various conditions (10 bar, 20 h).

To confirm whether NiO is the active phase of the catalyst, control experiments were performed by using non‐reduced NiO/Y and spent catalyst under various conditions. As shown in Figure 3a, the NiO/Y catalyst produced 0.11 mM_lactate_ under Ar, which is negligible compared to the 6.22 mM_lactate_ produced with Ni/Y. The 15.8% conversion can be attributed to the adsorption of reactants, as well as the possible decomposition of pyruvate by the acidic nature of zeolite Y. Given that insufficient H_2_ could lead to low lactate formation, a control experiment was conducted with NiO/Y catalyst under H_2_. This showed an improved lactate formation (0.44 mM), but the value was still much lower than that of the Ni/Y catalyst under Ar, suggesting that NiO was not the active phase of the catalyst for the reaction. Notably, a control experiment with the spent Ni/Y catalyst under an H_2_ environment showed 100% conversion and 14.1 mM_lactate_. This indicates that the spent catalyst was not deactivated, confirming that the fresh catalyst showed a plateau of conversion in 20 h due to insufficient H_2_ in the system. To investigate the role of Al in zeolite Y, a comparable Ni/SiO_2_ catalyst was tested under the same conditions. While fresh Ni/SiO_2_ exhibited higher conversion (64.2%), the spent catalyst showed no conversion even under H_2_ conditions, suggesting that Al plays a key role in pyruvate conversion (Figure S6).

To understand the dynamics of the reaction and the sigmoidal concentration‐time curve, the time‐dependent surface alteration of the catalysts was investigated using X‐ray photoelectron spectroscopy (XPS). In Figure 3b and Figure S7, the fresh catalyst consisted of 18.7% of Ni° and 81.3% of Ni^2+^. The high Ni^2+^ content is attributed to the surface NiO species formed during air passivation after reduction under H_2_ flow. After 2 h under the mild hydrothermal vent conditions of 100 °C and 10 bar_Ar_, there was no noticeable change in the surface Ni species, except for a slight decrease in Ni° (18.6%) and a slight increase in Ni^2+^ species (81.4%). This is consistent with the time‐resolved reaction profile in Figure 2d, where conversion and lactate formation were still less than 5% and 0.78 mM, respectively. However, extending the reaction time by 5 h resulted in the loss of surface metallic Ni species, leaving only oxidized Ni^2+^ species on the catalyst surface. At this point, the concentration‐time curve exhibited an exponential increase in conversion and lactate concentration to 30% and 3.2 mM, respectively, indicating that the catalyst was activated. The surface of the catalyst remained Ni^2+^ for the rest of the experiments, while the conversion and lactate formation rate were decreased due to the consumption of H_2_ for the hydrogenation of pyruvate. This resulted in a plateau of conversion and lactate formation, although the catalyst was still active.

Further investigation was carried out by comparing the XRD patterns of the catalysts after the reactions under different conditions. In Figure 3c, the XRD patterns revealed that the reflections from zeolite Y remained intact after the reactions, which means that the crystal structure of zeolite is stable under mild hydrothermal vent conditions. However, it was noted that the crystal structure of Ni was changed under some experimental conditions. When the 500 µmol_Ni_ was used as a catalyst under Ar, the reflections of Ni(111) and Ni(200) remained, indicating the bulk crystal structure of metallic Ni was retained. Subsequently, when the amount of Ni catalyst was decreased to 38 µmol under Ar, the reflections of Ni disappeared, indicating that metallic Ni was oxidized as observed in the XPS results. However, the reflections of NiO were not observed in the XRD pattern, suggesting that the phase formed could be amorphous or metallic Ni could be dissolved under hydrothermal conditions. The inductively coupled plasma optical emission spectroscopy results revealed that the reaction liquid contained 6.06 ppm of Ni, indicating a small amount of leaching during the reaction. Given that dissolved Ni species could act as a homogeneous catalyst for the hydrogenation of pyruvate, a control experiment with hot filtration was conducted. After 2 h of reaction with Ni/Y at 100 °C, the solid catalyst was separated from the liquid while it was still hot, and the solution remained under the reaction conditions for 18 h. The result showed that the leached Ni species did not contribute to the hydrogenation of pyruvate, which was supported by the lower concentration of lactate (1.1 mM) compared to that of Ni/Y (6.2 mM_lactate_), as shown in Figure S8. Similar results have been reported for transfer hydrogenation using homogeneous Ni catalysts with H_2_O as the hydrogen source, where Ni^2+^ in the form of NiCl_2_ showed no activity in the hydrogenation of alkenes and alkynes.^[^ ^49^, ^50^ ^]^

**Figure 4:**
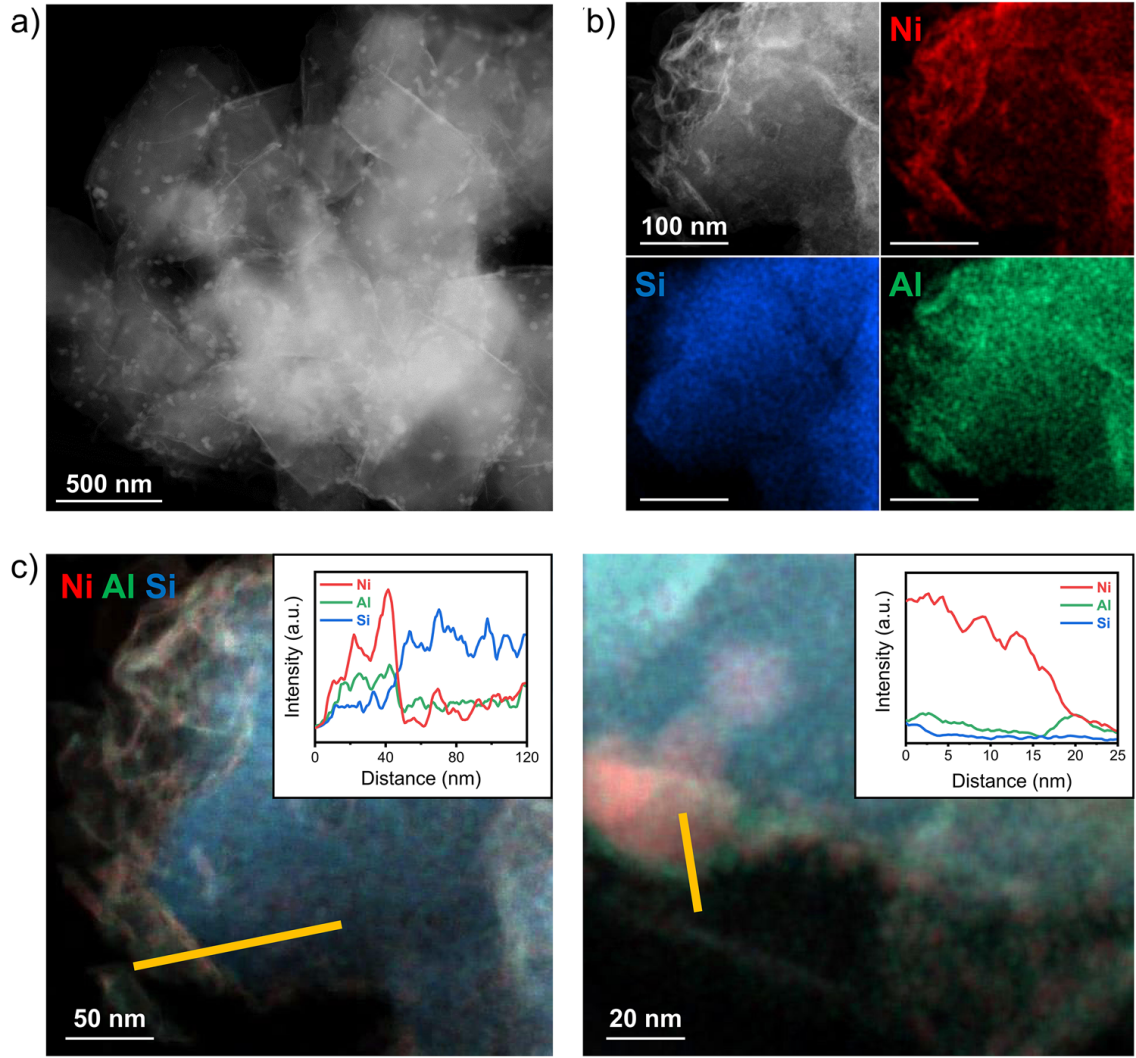
a) HAADF‐STEM image and b) EDX elemental mapping of Ni/Y catalyst after reaction at 100 °C for 20 h. c) Color‐combined images for line scan. The insets show the line scan profiles.

The spent catalyst was further investigated using HAADF‐STEM to gain further insight into understanding the alteration of the catalyst under mild hydrothermal vent conditions. As shown in Figure 4a, the morphology of zeolite Y was retained after the reaction under hydrothermal vent conditions. However, it was observed that Ni nanoparticles were homogeneously dispersed on zeolite Y, which contradicts the XRD and XPS results. To gain deeper insights into the spent catalyst, high‐magnification imaging and elemental mapping via EDX were conducted. The HAADF‐STEM image (Figure 4b) revealed the presence of thin surface layers on the spent catalyst, which were absent in the fresh sample. Elemental mapping confirmed that these layers primarily consisted of Ni, Al, and O, suggesting the leaching of Al from the zeolite Y framework. This phenomenon is attributed to the dealumination of zeolite Y, where hydrothermal treatment induces the extraction of aluminum from the framework structure.^[^ ^51^, ^52^ ^]^ The leached Al can form various extra‐framework Al species, such as Al^3+^, AlO^+^, Al(OH)^2+^, Al(OH)^2+^, AlO(OH), Al(OH)_3_, and Al_2_O_3_.^[^ ^53^, ^54^ ^]^ In addition, it was also found that some Ni nanoparticles with a large particle size of 40 nm were encapsulated by Al due to their strong interaction.

**Figure 5:**
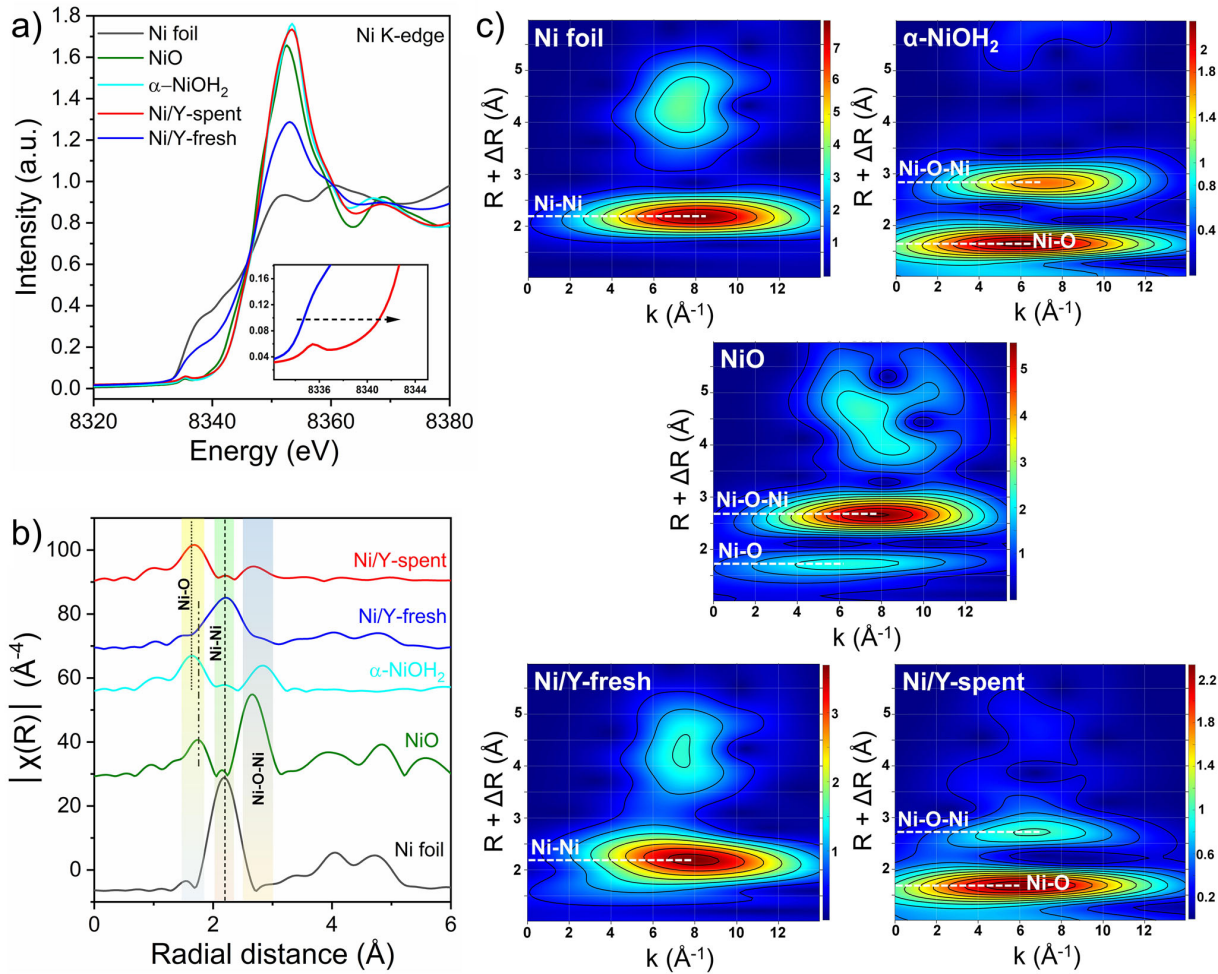
a) Experimental Ni K‐edge XANES spectra and b) experimental Ni K‐edge FT‐EXAFS spectra of Ni/Y‐fresh and Ni/Y‐spent with other control samples. c) WT‐EXAFS of Ni/Y‐fresh and Ni/Y‐spent with other control samples at Ni K‐edge.

To better understand the electronic structure and local coordination environment of Ni before and after catalysis, we employed X‐ray absorption spectroscopy (XAS). The Ni K‐edge X‐ray absorption near‐edge structure (XANES) of Ni/Y‐fresh closely matched that of Ni foil, indicating the metallic state of Ni on the zeolite Y support, in agreement with the XRD results (Figure 5a). Compared to Ni/Y‐fresh, the Ni K‐edge XANES spectra of Ni/Y‐spent exhibited a significant positive energy shift, aligning with the spectral features of NiO and α‐Ni(OH)_2_. This suggests a phase transformation from metallic Ni to oxidized Ni^2+^ during catalysis. The transformation was further confirmed by Ni K‐edge Fourier‐transformed k^3^ weighted extended X‐ray absorption fine structure (FT‐EXAFS) analysis, which provided detailed information on the coordination environment of the absorbing Ni center.

For Ni/Y‐fresh, the primary FT‐EXAFS peak at 2.2 Å was attributed to metallic Ni─Ni scattering. However, in the case of Ni/Y‐spent, the FT‐EXAFS spectra were dominated by peaks at 1.7 and 2.7 Å, corresponding to Ni─O and Ni─O─Ni scattering, respectively. These features are characteristic of NiO/α‐Ni(OH)_2_, further corroborating the transformation of metallic Ni to amorphous NiO/α‐Ni(OH)_2_ on the zeolite Y support after catalysis (Figures 5b and S9). In addition, Ni K‐edge wavelet transform‐EXAFS (WT‐EXAFS) analysis of Ni/Y‐spent (Figure 5c) revealed contributions consistent with Ni─O and Ni─O─Ni scatterings, providing further evidence of the phase transformation. These findings are consistent with other characterization techniques, confirming the successful transition from metallic Ni to oxidized Ni species during the catalytic process under hydrothermal vent conditions.

**Figure 6:**
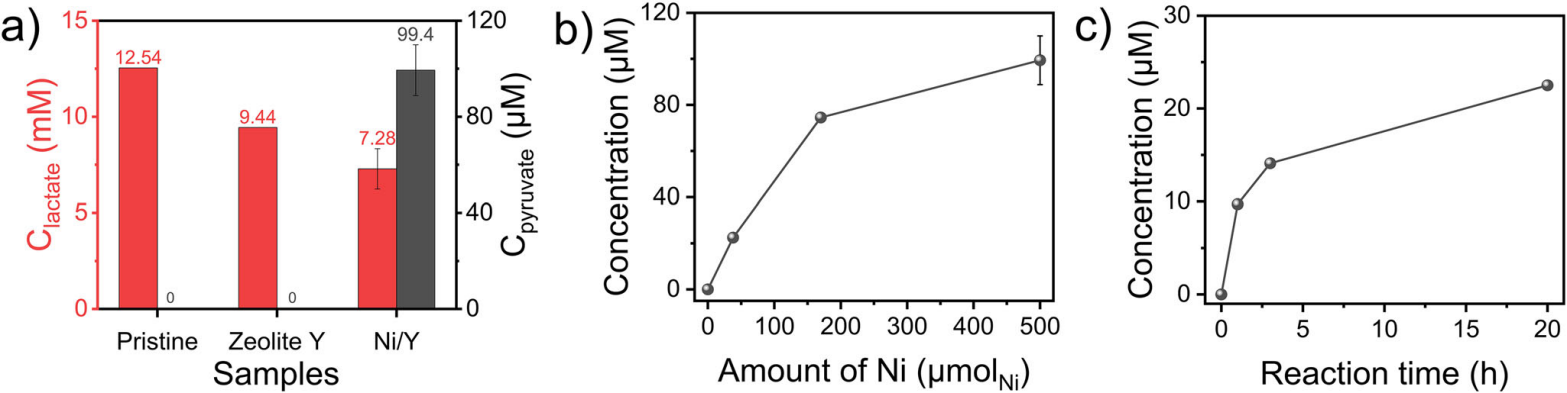
a) Control experiments for lactate conversion to pyruvate on zeolite Y (264 mg) and Ni/Y (293 mg). The concentration of pyruvate with respect to b) the amount of Ni for 20 h and c) the reaction time with 38 µmol_Ni_. Error bars represent the standard deviation from triplicate. Reaction conditions: 25 °C, atmospheric conditions, 20 h.

Moreover, the Ni/Y catalyst was further investigated for the conversion of lactate back to pyruvate in order to fully understand its activity under hydrothermal vent conditions. The conversion of lactate to pyruvate requires oxidizing conditions, which can be created in the mixing zone at hydrothermal vents.^[^ ^55^ ^]^ The presence of aerobic bacteria in hydrothermal vents further supports the potential for aerobic conditions.^[^ ^56^ ^]^ To mimic these conditions, although the oxidation potential in the ancient hydrothermal vent is not well defined, the conversion of lactate was carried out at 25 °C under atmospheric conditions as a proof of principle. The exposure of the catalyst to air does not affect its chemical properties due to the surface passivation procedure during preparation, but it facilitates the oxidative dehydrogenation of lactate to pyruvate.^[^ ^40^ ^]^ As shown in Figure 6a, the pristine sodium lactate solution showed no change for 20 h without a catalyst, indicating that lactate is stable at 25 °C under atmospheric conditions. The addition of the zeolite Y to the sodium lactate solution (12.54 mM_lactate_) exhibited a lower lactate concentration of 9.44 mM due to the adsorption of lactate on the microporous zeolite structure as shown in Figure S3. It was observed that the adsorption of lactate did not lead to the dehydrogenation of lactate since pyruvate was not detected. However, the addition of Ni/Y catalyst to sodium lactate solution produced 99.4 µM_pyruvate_ after 20 h under atmospheric conditions. This indicates that the dehydrogenation of lactate to pyruvate is driven by Ni sites. In Figure 6b, different amount of catalyst was screened, showing higher amount of Ni produced more pyruvate concentration. The investigation of the reaction time in Figure 6c exhibited that pyruvate was produced already after 1 h, reaching 9.7 µM_pyruvate_. The pyruvate concentration increased to 14.1 µM in 3 h and eventually reached 22.5 µM in 20 h. The results reflect that pyruvate, which was synthesized using transition metal minerals such as Ni°, Ni_3_Fe, and CoFe under hydrothermal vent conditions, can be interconverted to lactate using the single Ni/Y catalyst depending on the hydrothermal vent conditions. In biology, this process is carried out by LDH and NADH, which are complex organic molecules. This is an indication that the Ni/Y catalyst could pave the way for the biological interconversion of pyruvate and lactate, as well as the reverse conversion.

Finally, a reaction mechanism for pyruvate–lactate interconversion is proposed, although determining the exact mechanism under hydrothermal vent conditions remains challenging due to the dynamic nature of the catalyst surface and the presence of solvents. Some studies have examined this reaction under limited conditions using DFT calculations. Jones et al. reported the hydrogenation mechanism of pyruvic acid to lactic acid on a Cu{110} model surface using DFT calculations.^[^ ^57^ ^]^ They identified two possible reaction pathways for the hydrogenation of pyruvic acid to lactic acid under ultrahigh vacuum conditions in the gas phase. The first pathway begins with pyruvic acid binding to the surface while retaining its acidic hydrogen, which then transfers to the surface. Following this, hydrogen is sequentially added to the carbonyl oxygen and carbonyl carbon, leading to the formation of anionic lactate. An additional hydrogen from the surface is required to yield the final product, adsorbed lactic acid. The second pathway differs, with trans pyruvic acid binding to the surface and an internal hydrogen transfer from the acid group to the carbonyl oxygen. This pathway is more favorable than the first one due to fewer transition states. Therefore, under our reaction conditions, pyruvate may bind to the surface of nickel, and hydrogen transfer from ‐OH to C═O could take place, although this result is not directly applicable to our system. Regarding the reverse conversion of lactate to pyruvate, Yin et al. studied the reaction mechanism on α‐Fe_2_O_3_ using DFT simulations.^[^ ^58^ ^]^ They found that the iron species interacts with the ‐OH group of the lactic acid due to its lowest adsorption energy, suggesting that lactic acid is adsorbed and activated at the Fe^3+^ site. Subsequently, the O─H bond is broken, and a proton is transferred to the adjacent oxygen atom, forming a new Fe─OH group. The Fe─OH species then attacks the C─H bond, resulting in the formation of pyruvate. Meanwhile, the dissociated proton combines with Fe─OH to form water, and Fe^3+^ is reduced to Fe^2+^. Similarly, the oxidized nickel species in Ni/Y, as confirmed by XPS, may interact with the hydroxyl group of lactic acid to form Ni─OH species. These Ni─OH species could then engage with the C─H bond to facilitate pyruvate formation.

## Conclusion

We synthesized Ni/Y, which naturally occurs at hydrothermal vents, to investigate its role in the conversion of the metabolically crucial pyruvate under mild hydrothermal vent conditions. Our study demonstrates that Ni/Y can catalyze the conversion of pyruvate to lactate under these conditions, particularly in an inert gas environment. This suggests that H₂ is generated in situ through an autocatalytic reaction between Ni/Y and H₂O. A systematic investigation revealed a sigmoidal increase in lactate concentration, further supporting the autocatalytic nature of the process. Post‐reaction analysis using XAS and HAADF‐STEM identified the formation of amorphous nickel oxide and hydroxide, as well as the leached aluminum species from zeolite, which contributed to the activity. In oxidizing conditions similar to those found in the mixing zones of hydrothermal vents, the Ni/Y catalyst demonstrated the ability to reversibly convert lactate to pyruvate, providing a valuable proof of principle. This interconversion parallels the biological process catalyzed by LDH with NAD⁺ cofactors. Our findings highlight the potential of Ni/Y catalysts to drive the pyruvate–lactate interconversion under mild prebiotic conditions, suggesting that similar catalytic processes could have played a pivotal role in the emergence of life, laying the groundwork for the complex biochemical reactions mediated by enzymes and organic cofactors in modern biology.

## Supporting Information

The authors have cited additional references within the Supporting Information.^[^ ^59^ ^]^

## Conflict of Interests

The authors declare no conflict of interest.

## Supporting information



Supporting Information

## Data Availability

The data that support the findings of this study are available from the corresponding author upon reasonable request.
