# Supplementary material for: Prebiotic Interconversion of Pyruvate and Lactate over Zeolite‐Supported Ni Catalyst
Source: Angew Chem Int Ed Engl. 2025 Apr 21;64(23):e202503747. doi: 10.1002/anie.202503747 (PMC12124343; doi:10.1002/anie.202503747)
Supplement: Supplementary file 1 — Supporting Information [file ANIE-64-e202503747-s001.pdf]

Supporting Information
©Wiley-VCH 2021
69451 Weinheim, Germany

Prebiotic Interconversion of Pyruvate and Lactate over Zeolite-Supported Ni Catalyst

Y. Song, E. Budiyanto, A. Kumar, G. Landrot and H. Tüysüz*

Table of Contents

Experimental Procedures

Materials 3

Catalysts preparation 3

Characterization 3

Catalytic tests 3

Results

Figure S1. The XRD patterns of zeolite Y, calcined Ni/Y, and fresh Ni/Y catalyst after reduction. 4

Figure S2. Control experiments under various conditions with sodium pyruvate solution (13 mM), zeolite Y: (264 mg), Ni/Y (293 mg, 500 μmolNi), 10 bar, 20 hours. 4

Figure S3. The concentration of lactate regarding the amount of zeolite Y. Adsorption condition: sodium lactate solution (12.5 mM, 3 mL), 25 ºC, atmospheric condition, 20 hours. 5

Figure S4. The ^{13}C-NMR spectrum after 20 hours at 100 ºC under 10 bar_{Ar} with 38 $μmol_{Ni}$ of Ni/Y catalyst with sodium pyruvate-2-^{13}C. δ(ppm)= 205.08 (pyruvate-2-^{13}C), 68.65 (lactate-2-^{13}C). 5

Figure S5. The effect of Ar pressure on the conversion and lactate concentration. Reaction conditions: Ni/Y (38 $μmol_{Ni}$), 100 ºC, 20 hours. 6

Figure S6. (a) XRD patterns of fresh and spent Ni/SiO_2 catalyst. (b) Catalytic performance investigation under various conditions (38 $μmol_{Ni}$, 100 ºC, 10 bar, 20 hours). (c) TEM images of fresh (left) and spent (right) Ni/SiO_2 catalyst. 6

Figure S7. The surface composition of Ni from XPS Ni 2p spectra. Reaction condition: Ni/Y (38 $μmol_{Ni}$), 100 ºC, 10 bar_{Ar}, 20 hours. 7

Figure S8. Time-resolved lactate concentration with Ni/Y (38 $μmol_{Ni}$, red) and hot filtration after 2 hours (blue). 7

Figure S9. Experimental Ni K-edge EXAFS spectra of Ni/Y-fresh and Ni/Y-spent with other control samples. 8

Experimental Procedures

Materials

Nickel nitrate hexahydrate ($Ni(NO_3)_2{\cdot}6H_2O$, 98%), sodium pyruvate (99%), sodium pyruvate-2-^{13}C (99% ^{13}C), sodium lactate, and silica gel (Davisil Grade 62, pore size 150 Å, 60-200 mesh) were purchased from Sigma Aldrich. Zeolite Y, hydrogen (730 m^2/g, 5.1:1 mole ratio SiO_2:Al_2O_3) was purchased from Alfa Aesar.

Catalysts preparation

Zeolite-supported Ni (Ni/Y) was prepared by wet impregnation method. Typically, $Ni(NO_3)_2{\cdot}6H_2O$ (4.954 g) was dissolved in milli-Q water (50 mL) with magnetic stirring. Zeolite Y (9 g) was added to the solution to achieve 10 wt% Ni loading. The mixture was dried at 100 °C overnight. The resulting powder was calcined at 350 °C for 3 hours with a ramping of 5 °C/min under static air, followed by reduction at 400 °C for 3 hours with a stream of 10% H_2 in Ar (100 mL/min). The catalyst was passivated for 30 minutes with a stream of 10% air in Ar (100 mL/min) at room temperature. Ni/SiO_2 was synthesized using the same procedure, with silica gel as the support material.

Characterization

The powder X-ray diffraction (XRD) patterns were analyzed using a PANalytical X'Pert diffractometer with Cu radiation (K_α= 1.541874 Å). The instrument was equipped with a K_β-Filter and an RTMS X'Celerator Scientific detector. Data collection was performed in the range of 2θ from 5 to 90° with a step width of 0.0167° 2θ, and measured for 100 seconds per step. Ten scans were collected for each sample and summed post-collection. Samples were mounted on a silicon background-free holder for the measurements. The obtained patterns were evaluated by comparing them with entries from the International Centre for Diffraction Data PDF-2. High-angle annular dark field scanning transmission electron microscopy (HAADF-STEM) images were acquired on Talos F200X (Thermo Scientific) with 200 kV. Samples were loaded on a Cu lacey carbon grid (400 mesh) through dry deposition. Energy-dispersive X-ray spectroscopy (EDX) was carried out on a Super-X EDS system equipped with four silicon drift detectors and analyzed on Velox software. X-ray photoelectron spectroscopy (XPS) was conducted with a custom spectrometer from SPECS GmbH, featuring a PHOIBOS 150 hemispherical energy analyzer and a 1D-DLD detector. The monochromatized Al K_α X-ray source (E= 1486.6 eV) was operated at 15 kV and 200 W in medium area mode. During the experiments, the base pressure in the analysis chamber was 5 × 10^{-10} mbar. To correct for charging effects, all spectra were referenced to the C1s signal at 284.5 eV. High-performance liquid chromatography (HPLC) was performed using Shimadzu LC-2030C with a RID-20A refractive index detector. Separations utilized a 300 x 6.5 mm Agilent MetaCarb 67H column with a guard column, and 0.1% trifluoroacetic acid in water as the mobile phase, at a flow rate of 0.8 mL/min and a temperature of 323K. Results were quantified using a single-point external calibration for each compound. The nuclear magnetic resonance (NMR) spectrum was recorded on a Bruker Avance II spectrometer, operating at a field of 14.1T (^1H Larmor frequency of 600 MHz) and equipped with cryogenically cooled TCI (inverse) probe heads for the maximum sensitivity in direct-observation 1H. All spectra were recorded at 298K in standard 3 mm tubes containing sample volumes of 270 µL and sodium trimethylsilylpropanesulfonate (DSS) dissolved D_2O (1.67 mM, 30 µL). In the ^1H spectrum, water suppression at 4.68 ppm was achieved using excitation sculpting combined with a perfect echo, utilizing the Bruker standard pulse program zgesgppe. Data acquisition involved 1024 transients (ns), totaling 80 minutes of experimental time. All spectra were analyzed with MNova 14. Inductively coupled plasma-optical emission spectrometry (ICP-OES) samples were analyzed on SPECTROGREEN, SPECTRO Analytical Instruments. Ni K-edge X-ray absorption spectroscopy (XAS) measurements were conducted in transmission mode. The experiments were performed at the SAMBA beamline of the SOLEIL Synchrotron, operating with an electron beam current of 450 mA. Powder samples were prepared by homogeneously mixing the respective material with cellulose, compressing the mixture into pellets (diameter = 13 mm), and sealing them with 30 µm Kapton tape. A Si(220) double-crystal monochromator was employed for energy selection, providing an incident photon flux of approximately 7.5 x 1011 ph/s with a beam size of 2 x 0.5 mm. Energy calibration was conducted during each measurement using a metallic reference placed before the third ion chamber. The incident energy was calibrated to the inflection point of the Ni K-edge in the metallic foil, set at 8333.0 eV. The acquired spectra were processed and normalized using the Fastosh program.[59]

Catalytic tests

Autoclaves made of C-276 alloy were used for catalytic experiments. Ti liners were chosen to avoid contamination from the reactor. The reaction volume with Ti liner is 20 mL (ϕ 2.3 cm×5 cm). A polyetheretherketone ring is used for secured sealing. Blank tests did not exhibit product, confirmed by HPLC and ^1H-NMR. For typical experiments, Ni/Y catalyst was added to the Ti-lined autoclave with sodium pyruvate or lactate solution (13 mM, 3 mL) prepared by dissolving sodium pyruvate or lactate in Milli-Q water. After the autoclave was sealed with 16 N·m, the chamber was purged at least three times with the desired gas and pressurized to 10 bar at room temperature. The autoclave was heated to the target temperature with an electric heating system. After the reaction, the autoclave was cooled down in an ice bath. The liquid was collected by syringe filtration (PTFE, pore size of 0.2 µm) for HPLC and ^1H-NMR analyses. Catalysts were collected by filtration and dried at 60 °C under vacuum. Dried catalysts were analyzed by XRD, XPS, and TEM.

Results

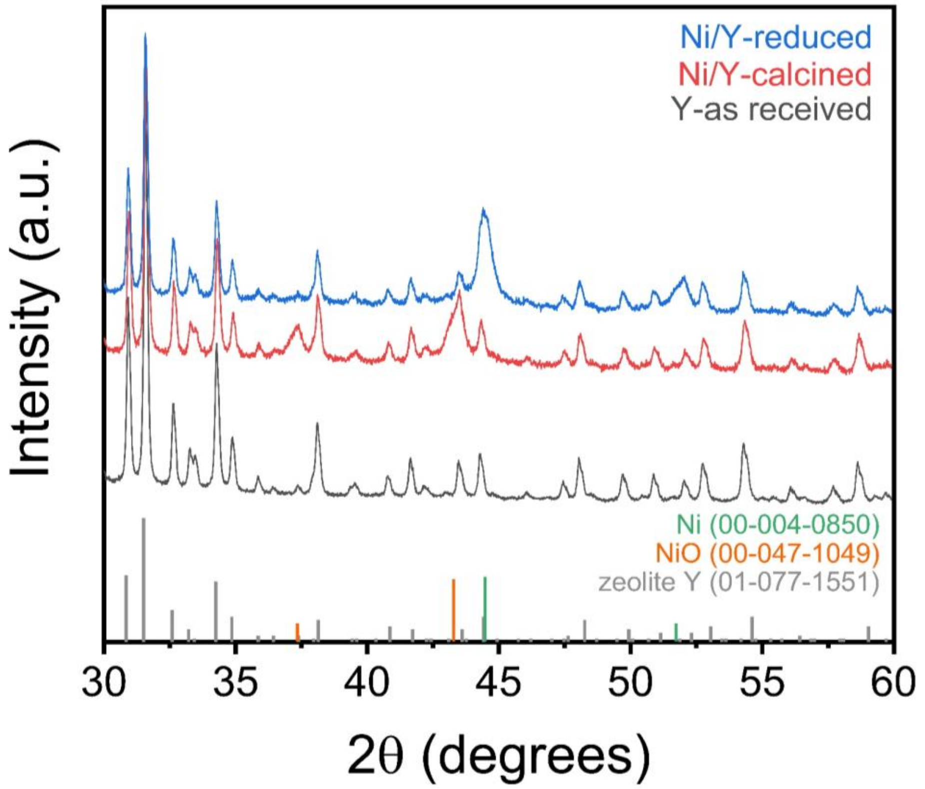

Figure S1. The XRD patterns of zeolite Y, calcined Ni/Y, and fresh Ni/Y catalyst after reduction.

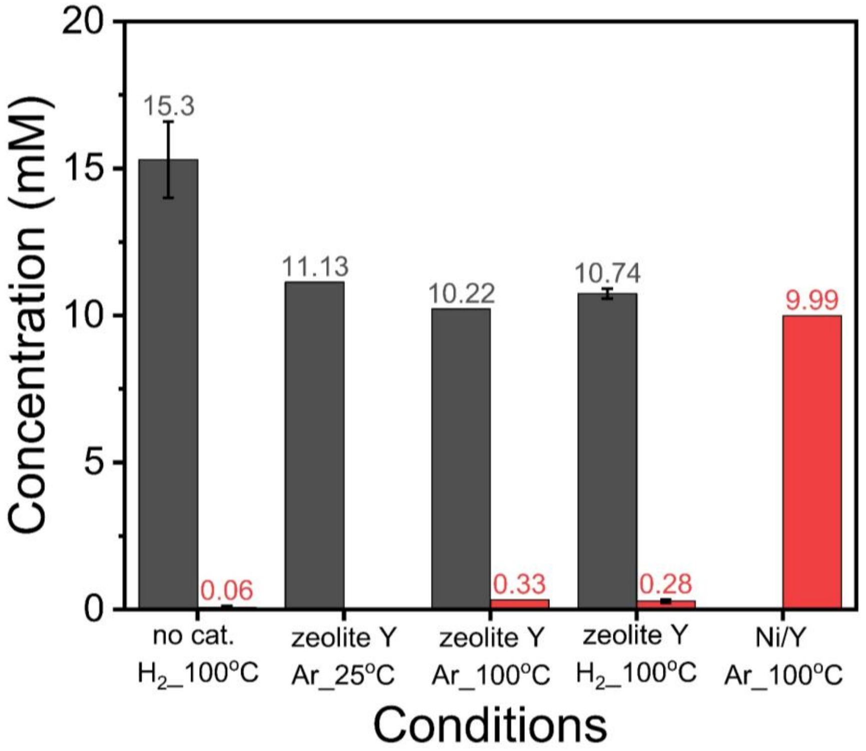

Figure S2. Control experiments under various conditions with sodium pyruvate solution (13 mM), zeolite Y: (264 mg), Ni/Y (293 mg, 500 μmol_{Ni}), 10 bar, 20 hours.

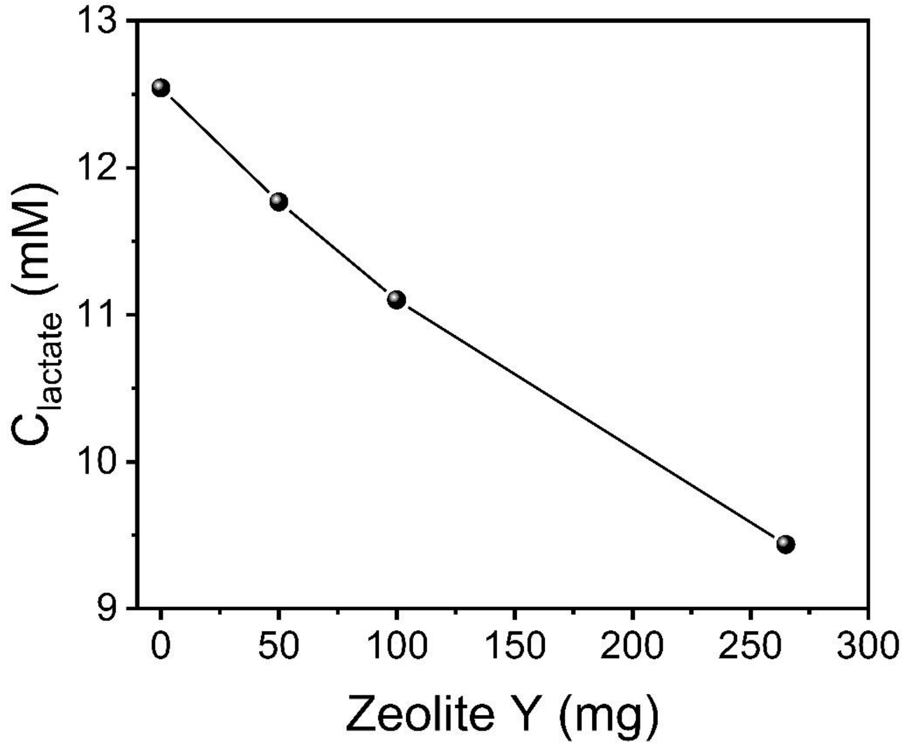

Figure S3. Lactate concentration as a function of zeolite Y amount (0, 50, 100, and 264 mg). The corresponding Ni content in Ni/Y was calculated as 0, 85, 170, and 500 μmol for comparison with Fig. 2c. Adsorption condition: sodium lactate solution (12.5 mM, 3 mL), 25 °C, atmospheric condition, 20 hours.

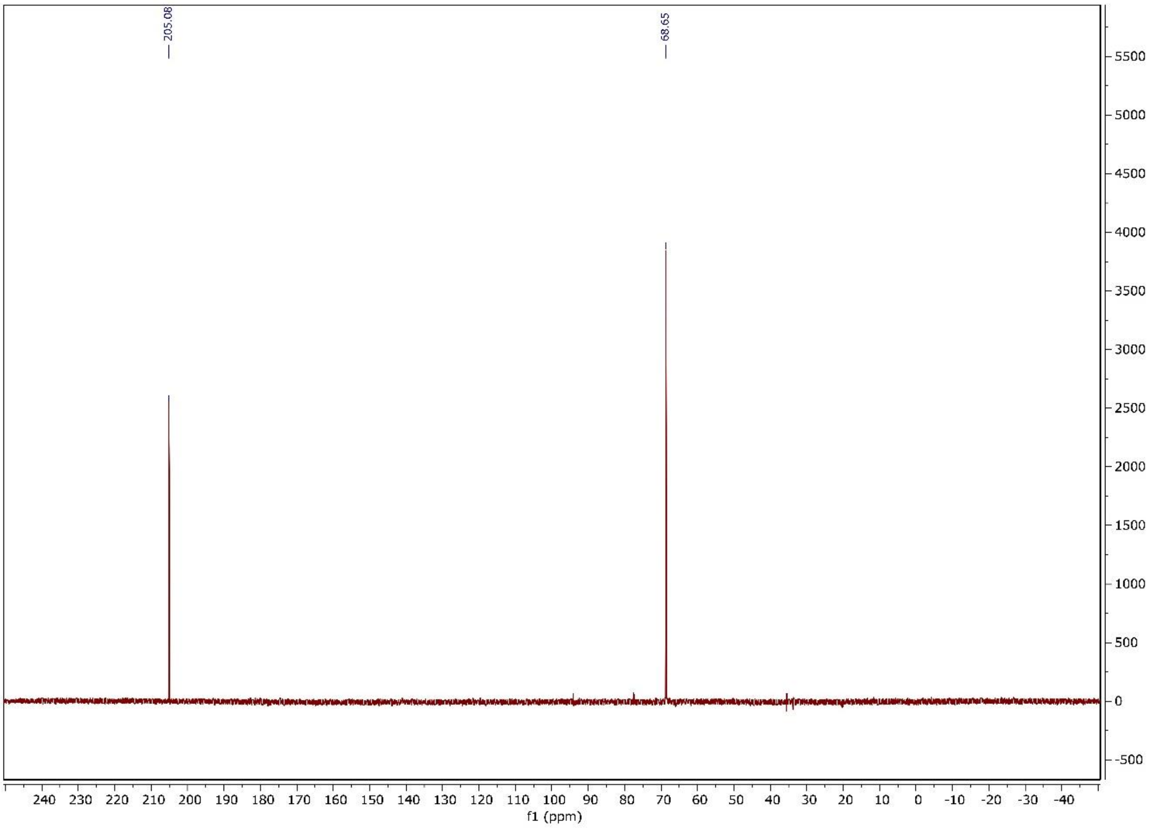

Figure S4. The ^{13}C-NMR spectrum after 20 hours at 100 °C under 10 bar_{Ar} with 38 μmol_{Ni} of Ni/Y catalyst with sodium pyruvate-2-^{13}C. δ(ppm)= 205.08 (pyruvate-2-^{13}C), 68.65 (lactate-2-^{13}C).

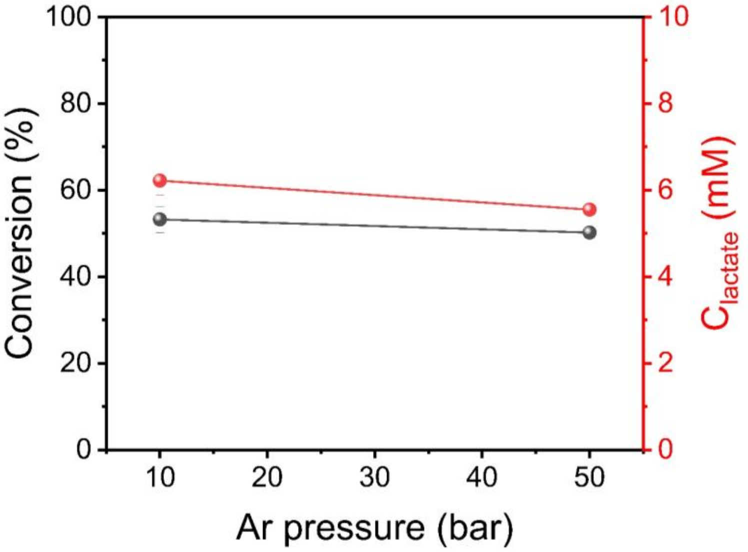

Figure S5. The effect of Ar pressure on the conversion and lactate concentration. Reaction conditions: Ni/Y (38 μmol$_{Ni}$), 100 °C, 20 hours.

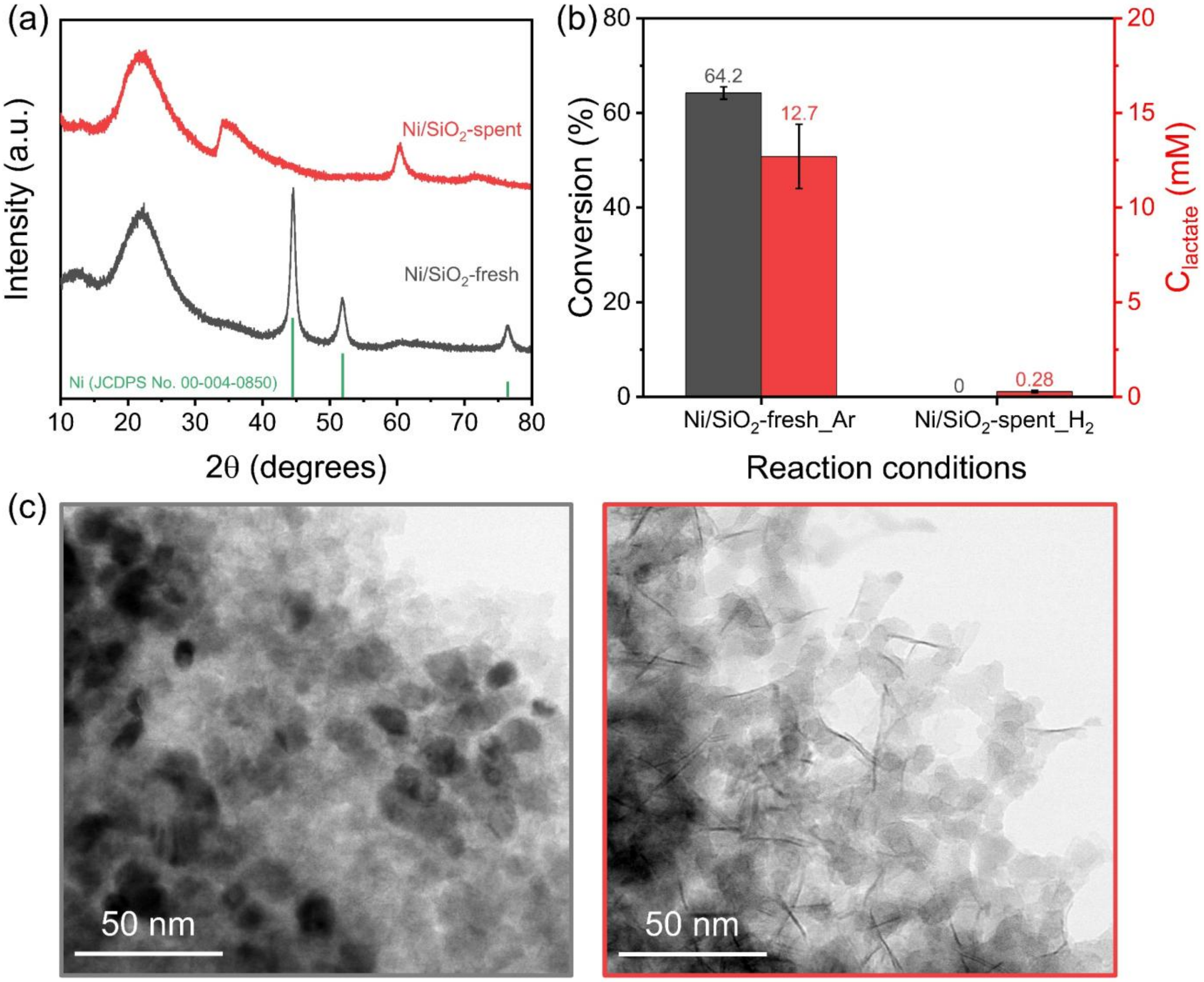

Figure S6. (a) XRD patterns of fresh and spent Ni/SiO$_2$ catalyst. (b) Catalytic performance investigation under various conditions (38 μmol$_{Ni}$, 100 °C, 10 bar, 20 hours). (c) TEM images of fresh (left) and spent (right) Ni/SiO$_2$ catalyst.

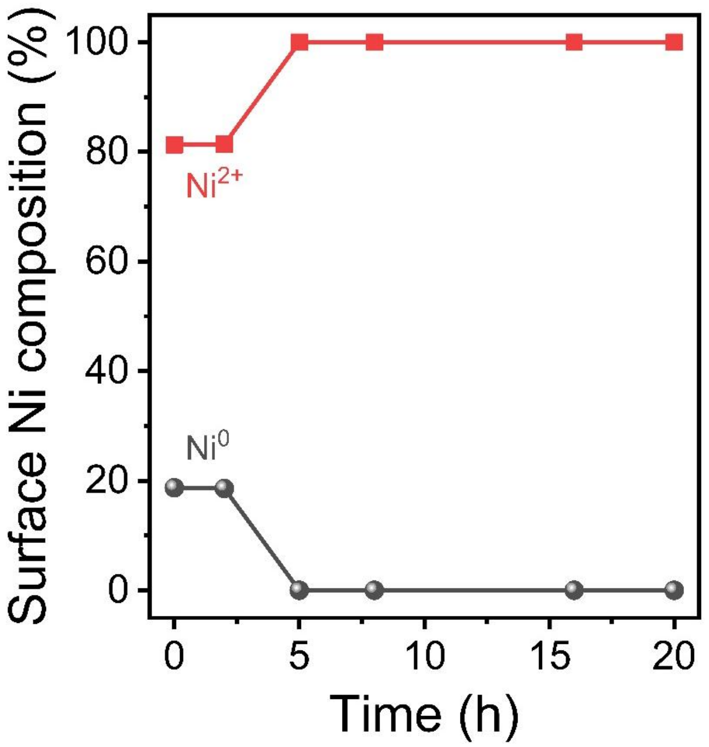

Figure S7. The surface composition of Ni from XPS Ni 2p spectra. Reaction condition: Ni/Y (38 μmol$_{Ni}$), 100 °C, 10 bar$_{Ar}$, 20 hours.

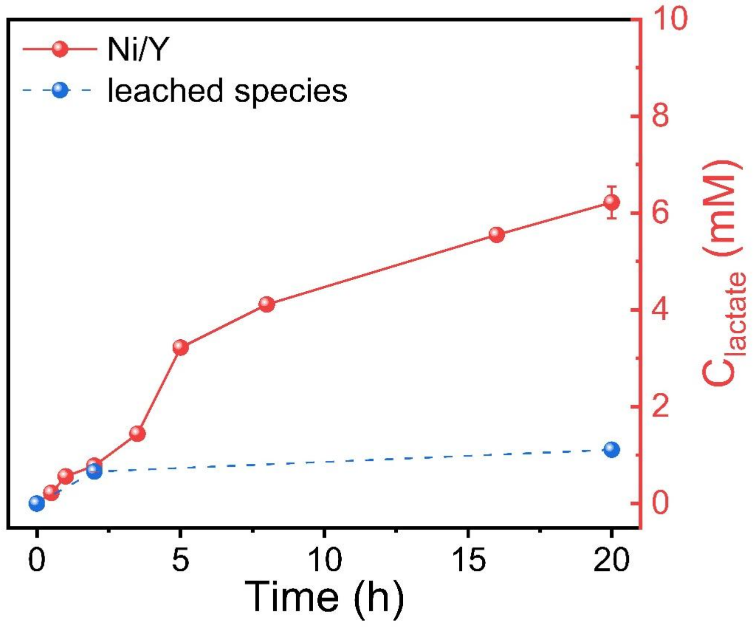

Figure S8. Time-resolved lactate concentration with Ni/Y (38 μmol$_{Ni}$, red) and hot filtration after 2 hours (blue).

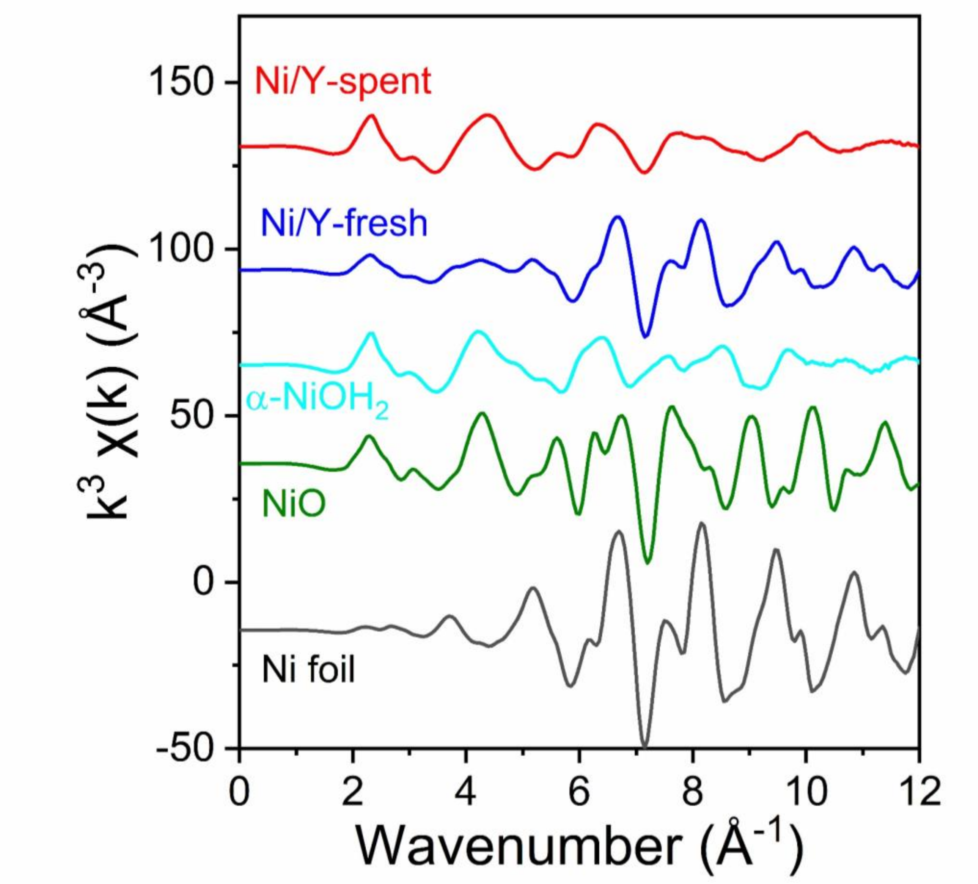

Figure S9. Experimental Ni K-edge EXAFS spectra of Ni/Y-fresh and Ni/Y-spent with other control samples.
